# The Icelandic 16-electrode electrohysterogram database

**DOI:** 10.1038/sdata.2015.17

**Published:** 2015-04-28

**Authors:** Asgeir Alexandersson, Thora Steingrimsdottir, Jeremy Terrien, Catherine Marque, Brynjar Karlsson

**Affiliations:** 1 Reykjavik University, School of Science and Engineering, Reykjavik 101, Iceland; 2 Landspitali University Hospital, Ob-Gyn department, Reykjavik 101, Iceland; 3 Université de Technologie de Compiègne, Biomécanique et Bio-ingénierie, Compiègne 60203, France

**Keywords:** Biomedical engineering, Databases, Electrophysiology, Electromyography - EMG

## Abstract

External recordings of the electrohysterogram (EHG) can provide new knowledge on uterine electrical activity associated with contractions. Better understanding of the mechanisms underlying labor can contribute to preventing preterm birth which is the main cause of mortality and morbidity in newborns. Promising results using the EHG for labor prediction and other uses in obstetric care are the drivers of this work. This paper presents a database of 122 4-by-4 electrode EHG recordings performed on 45 pregnant women using a standardized recording protocol and a placement guide system. The recordings were performed in Iceland between 2008 and 2010. Of the 45 participants, 32 were measured repeatedly during the same pregnancy and participated in two to seven recordings. Recordings were performed in the third trimester (112 recordings) and during labor (10 recordings). The database includes simultaneously recorded tocographs, annotations of events and obstetric information on participants. The publication of this database enables independent and novel analysis of multi-electrode EHG by the researchers in the field and hopefully development towards new life-saving technology.

## Background & Summary

Preterm birth is defined as birth before 37 completed weeks of gestation. On average, 12% of babies are born preterm in lower-income countries and 9% in higher-income countries^1^. Babies who are born preterm often require special care and face greater risks of serious health problems, including cerebral palsy, intellectual impairment, chronic lung disease, and vision and hearing loss. Preterm birth is the leading cause of newborn deaths (babies in the first four weeks of life) and the second leading cause of death in children under five years (after pneumonia). Preterm birth rates are increasing in almost all countries^1^.

Currently, there is no effective way of preventing preterm birth. The main reason is that no good objective method currently exists to evaluate the stepwise progression of pregnancy through to labor, neither at term nor preterm. Various techniques have been adopted to monitor and/or diagnose labor, but they are either subjective or indirect and do not provide an accurate prediction of when labor will take place^2–5^. Studies have shown that external monitoring of uterine electrical activity using an electrohysterogram (EHG) is representative of uterine contractility^6^ and show promising results in predicting preterm labor^3–5,7,8^. Most of the early studies on EHG used two to five electrodes^2,6^ and therefore concentrated on the activity in a single location of the uterus. In 2007, a collaborative group from France and Iceland, involving biomedical researchers, engineers and medical doctors, started using monopolar electrodes in a 4-by-4 configuration on the abdomen aimed at providing information on uterine electrical activity propagation^9^. Guided by this preliminary work, the 16-electrode system was used to perform pregnancy and labor recordings in Iceland at Landspitali University Hospital, Akureyri Hospital and the Akureyri Primary Health Care Centre. In total, a database of 122 EHG recordings was created between 2008 and 2010. The majority of participants were measured multiple times during the same pregnancy and took part in two to seven recordings. These multiple, or longitudinal, measurements were aimed at observing the evolution of contractions during pregnancy and towards labor.

Parts of the data have already been used for developing and analyzing various signal processing methods and have led to several publications^8,10–23^. In particular, the work has concentrated on efforts to accurately distinguish true labor contractions from normal pregnancy contractions, with some success^8,10–17,20–23^. Compared to linear methods, non-linear methods may provide a superior way to differentiate between pregnancy and labor contractions^8,11,22^ and multichannel recordings seem to improve this classification rate^10^. Interest in EHG propagation parameters has been increasing among researchers^24^ and efforts have been made to exploit the information on the propagation and/or synchronization of different locations of the uterus which the extended geometry of the measurement system provides. The results indicate that propagation parameters can be important in accurately recognizing true labor^25,26^. Parts of the data were included in the BioModUE_PTL project which led to the development and validation of a biophysics based multiscale model of the EHG, going from the cell to the electrical signal measured on the abdomen^27^. None of these past publications, however, have described the database in detail and studies have so far only used parts of the data presented.

We provide open access to the database so that the international scientific community can freely generate greater understanding of the mechanics of the uterus and develop applications that improve obstetric care and hopefully accurately predict preterm labor. This paper describes the recording methods used and gives a detailed description of the Icelandic 16-electrode electrohysterogram database.

## Methods

### Data collection

The recordings were performed between 2008 and 2010 in Iceland. Pregnancy recordings, defined as recordings performed at antenatal care clinics on participants in the third trimester and not suspected to be in labor, were performed at Akureyri Primary Health Care Centre and Landspitali University Hospital. Labor recordings, defined as measurements performed on participants suspected to be in labor, present in the labor wards and who delivered within 24 h, were performed at Landspitali University Hospital and Akureyri Hospital. Participants were invited to take part in the recordings during antenatal care visits or at the labor wards and had normal singleton pregnancies and no known risk factors for preterm birth. Informed consent was obtained from every participant and the protocol was approved by the National Bioethics Committee in Iceland (VSN 02-006-V4). After each pregnancy recording, the participant was invited to take part in another recording one to two weeks later. All data can be found in PhysioNet (Data Citation 1).

### Recording protocol

**Figure 1:**
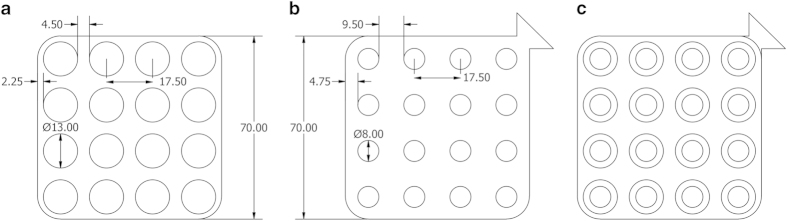
Silicone backing and double sided adhesive sheet. The dimensions (mm) of the silicone backing (**a**) and double sided adhesive sheet (**b**) are shown along with the back view of the silicone backing attached to the double sided adhesive sheet (**c**). The thickness of the silicone backing is 1.5 mm.

Reusable Ag/AgCl electrodes with a 13.0 mm outer diameter and an 8.0 mm inner diameter were used for the recordings. An alignment frame, a double sided hypoallergenic adhesive sheet and a silicone backing were designed and manufactured to enable a standardized electrode setup with a 17.5 mm distance between adjacent electrode centers. The alignment frame was used to align and attach an uncovered side of the double sided adhesive sheet to the silicone backing. The dimensions of the double sided adhesive sheet and silicone backing can be seen in Fig. 1 along with the back view of the silicone backing attached to the double sided adhesive sheet.

**Figure 2:**
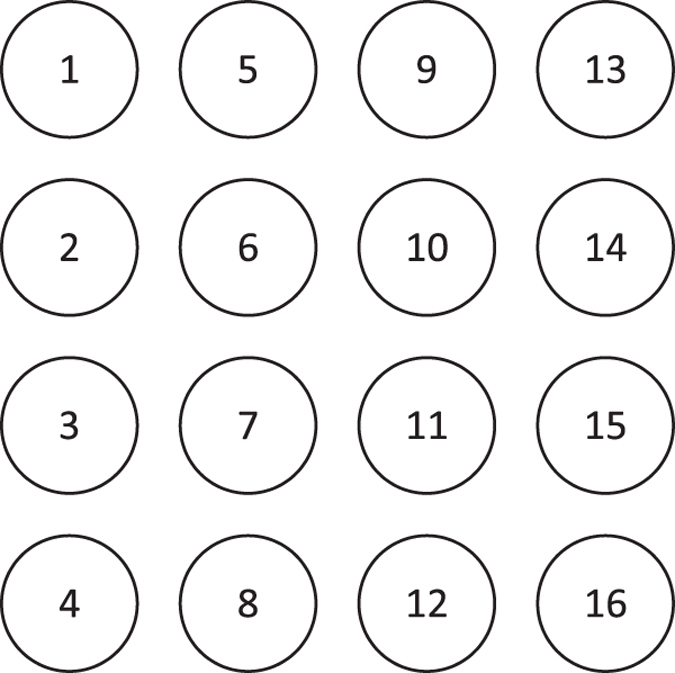
The electrode numbering scheme, as seen when looking at the abdomen of the participant.

The electrodes were then placed into the holes in the silicone backing and attached to the adhesive sheet. The abdominal skin of the participant was carefully prepared using an abrasive paste and alcohol solution. After filling the electrode holes with electrode gel, wiping the excess gel away with a straight edged card and uncovering the other side of the double sided adhesive sheet, the electrode-adhesive-silicone matrix was attached to the abdomen. The electrode numbering scheme, as seen when looking at the abdomen of the participant, can be seen in Fig. 2.

**Figure 3:**
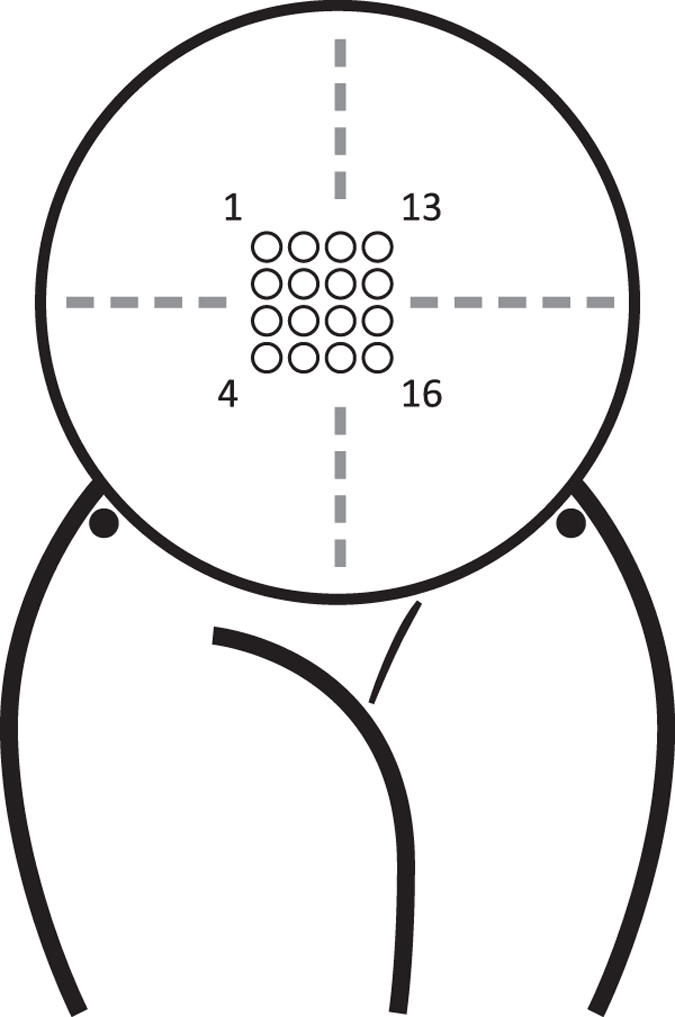
Ideal position of the 4-by-4 electrode grid. The third vertical line of electrodes (electrodes 9 to 12) is placed on the median axis of the uterus and the 10th–11th pair of electrodes half way between the uterine fundus and pubic symphysis. The corner electrodes of the 4-by-4 grid are labelled. The black electrodes represent the patient ground and the reference electrodes (sides not standardized).

The desired position on the abdomen was with the third vertical line of electrodes (electrodes 9 to 12) placed on the median axis of the uterus and the 10th–11th pair of electrodes half way between the uterine fundus and pubic symphysis. The navel was avoided by displacing the matrix up or down whilst staying as close as possible to the desired position. The skin over the iliac crests on both sides was prepared in the same way as the abdomen and a patient ground electrode and reference electrode with electrode gel were then attached on each side using adhesive washers, with inner diameters corresponding to the inner diameter of the electrodes. The locations of the reference and patient ground electrodes were not standardized to certain sides for the recordings. The electrode positions can be seen in Fig. 3.

**Figure 4:**
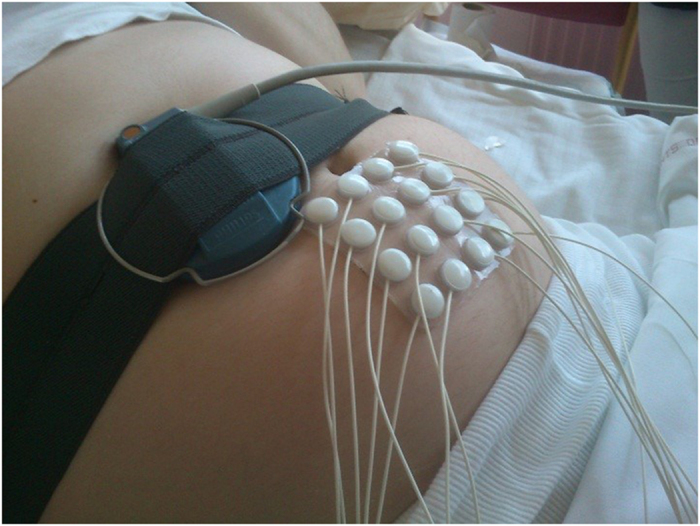
The recording setup. The abdominal electrodes and the tocodynamometer can be seen.

A tocodynamometer was also attached to the abdomen during recordings. For pregnancy recordings, the participants were seated in recliner chairs and a support, such as a small pillow, was positioned under the right side of the participants to prevent potential aortocaval compression syndrome. For labor recordings, the participants were lying on their beds in the maternity wards and the researcher did not try to affect their positioning. A photo of the setup during a recording can be seen in Fig. 4.

The intended duration of a pregnancy recording was one hour and the intended duration of a labor recording was at least half an hour, but the participant could stop the measurement at any time.

The measurements were performed using a sixteen channel multi-purpose physiological signal recorder (Embla A10), most commonly used for investigating sleep disorders. An anti-aliasing filter with a high cut-off frequency of 100 Hz was used but no high pass filter was used. The signal sampling rate was 200 Hz and the signal was digitized to 16 bits. The sixteen monopolar electrode signals were originally stored in the EDF (European Data Format) format by the Somnologica software used to control the Embla A10.

All the recordings were performed by the same person (A.A.). Each participant was assigned an ID number and for each recording, information on participant age, body mass index (BMI) gestational age, placental position, gravidity, parity, history of cesarean section, eventual mode of delivery and gestational age at delivery was noted. During a recording, the researcher recorded participant movements, equipment manipulation, participant-reported possible contractions and fetal movements, and any other unusual events. The participant and researcher conversed freely during the recordings and no restrictions were placed on the participant in terms of changing position if needed.

### Data processing

No processing of data was performed beyond converting between file formats. The aim is that those that use the data can do so with a fresh start. Future additions to this database may include pre-treated signals and segmented contractions.

The EDF files obtained during the recordings were converted into WFDB (WaveForm DataBase—www.physionet.org/physiotools/wfdb.shtml) compatible signal (.dat) and header (.hea) files using the edf2mit WFDB application (www.physionet.org/physiotools/wag/edf2mi-1.htm) and Cygwin software (cygwin.com). Annotation (.atr) files containing events during recordings were created manually by using the WFDB-compatible signal and header files and WAVE, an X Window System client application (www.physionet.org/physiotools/wfdb.shtml#WAVE). The wfdb2mat WFDB application (www.physionet.org/physiotools/wag/wfdb2m-1.htm) was used to convert the WFDB-compatible signal and header files into MATLAB (.mat) and corresponding header (.hea) files. The rdann function from the WFDB Toolbox for MATLAB (www.physionet.org/physiotools/matlab/wfdb-app-matlab) was used to create MATLAB (.mat) files containing the information from the annotation files. The tocograph paper traces were scanned to JPEG images and a time axis corresponding to the recording was inserted onto the scanned images. All information that could possibly lead to the identification of the participant, such as personal data and dates, was manually removed from these images using a graphics editor.

## Data Records

The data records in the Icelandic 16-electrode electrohysterogram database are stored in a PhysioBank database in PhysioNet^28^ (Data Citation 1).

A total of 122 recordings were performed on 45 participants. Of the 45 participants, 32 were measured more than once during the same pregnancy and the highest number of recordings for a participant was seven recordings. Ten recordings were performed during labor and five participants took part in measurements during both pregnancy and labor. The lowest gestational age was twenty nine weeks and five days (29w5d—pregnancy recording) and the highest gestational age was forty one weeks and five days (41w5d—labor recording). The average recording duration for pregnancy recordings was 61 min (range 19–86 min) and the average recording duration for labor recordings was 36 min (range 8–64 min).

File names in the database are of the form ice###_*type*_*record number*, where ice### is the ID of the participant (e.g., ice001), *type* refers to the type of recording: p (pregnancy) or l (labor), and *record number* is the number of recording for that particular participant (e.g., 1of3).

Each recording has three associated files:

-A scanned tocograph with a manually inserted recording time axis (.jgp file). Each small square represents 30 s.

-A binary signal (.dat) file containing the data from the 16 monopolar electrodes.

-A header (.hea) file:

The fields in the header files are according to the WFDB convention and are listed in detail on the PhysioNet website (www.physionet.org/physiotools/wag/header-5.htm). Lines 2–17 of the header files are signal specification lines and the strings at the end of these lines correspond to the signal labels. The signals are labelled with ‘EHGn’ where ‘n’ refers to the relevant electrode number (electrode numbers are not in ascending order).

Information from each recording is at the end of the header files and includes:

-Participant ID

-Record number

-Record type (labor, pregnancy)

-Age of participant (years)

-BMI (body mass index) of participant before pregnancy

-BMI of participant at time of recording

-Gravidity (number of times participant has been pregnant, including current pregnancy)

-Parity (previous births after 22 weeks gestation)

-Previous cesarean (Yes, No)

-Placental position

-Gestational age at recording (weeks/days), according to a first trimester ultrasound

-Gestational age at delivery (weeks/days),

-Mode of delivery (Vaginal, Vaginal/Induction, Elective cesarean, Emergency cesarean due to slow progress, Emergency cesarean due to other than slow progress). Vaginal delivery indicates spontaneous onset unless appended with /Induction.

-Synthetic oxytocin use in labor (Yes, No)

-Epidural during labor (Yes, No)

-Comments for recording

-Comments for delivery

For 111 of the recordings, there is also an annotation (.atr) file containing the type and timing of events.

The types of event are:

- C—Contraction. Used when the participant feels a contraction or there is a very likely contraction on the tocograph (not always used when there is an obvious contraction on the tocograph).

- (c)—Possible contraction. Used when there is not a very likely contraction but the participant has pressure sensation or a contraction is suspected on the tocograph.

- pm—Participant movement.

- pos—Participant change of position.

- fm—Fetal movement. Used when the participant feels fetal movement.

- em—Equipment manipulation. Used when electrodes are pressed more firmly onto the abdomen if otherwise not explained in the comments.

The database also includes:

-The zip file icelandic16ehgmat.zip that includes MATLAB (.mat) versions of all the signal files along with header files (file names of the form ice###_*type*_*record number*m) and MATLAB (.mat) versions of the annotations (file names of the form ice###_*type*_*record number*m_ann). This is provided for the convenience of users that want to analyze the data in MATLAB.

-RECORDS.txt containing a list of the recordings by record name, with one record name per line.

Table 1 (available online only) contains the clinical information from each recording (information from the header files excluding comments) along with the recording duration and whether or not the recording has a corresponding annotation file. This information can also be found in info.txt in the database.

## Technical Validation

The EHG signal has been shown to be representative of uterine contractions^6^ and EHG is, in general, a well-proven technique^5^. The proof of concept and technical validation for the recording method used for the database was made in a preliminary study in 2007 (ref. 9).

The preliminary study applied recognized EHG techniques to a new recording setup involving a 4-by-4 grid. This was mainly to better observe and analyze the spatial characteristics and propagation of the electrical activity during contractions rather than just the activity in a single location of the uterus as had been done before^2,6^. The results from the preliminary study showed a very acceptable SNR (signal to noise ratio) of bipolar signals (difference between adjacent monopolar electrodes)^29^. The use of the monopolar signals singly was however considered problematic, even with adaptive filtering methods due to external common mode noise (maternal ECG, respiration movements etc.). Efforts to use recent techniques such as empirical mode decomposition (EMD) and canonical component analysis (CCA) to clean up the signal have since met with some success^30^. The preliminary study data was also used to present a moving picture of the electrical activity during contractions. The activity was clearly observed and correlated well with the simultaneous tocograph trace^9^.

In the preliminary study, electrodes were placed one at a time for the 4-by-4 grid, which was a time-consuming task and achieving the desired inter-electrode distance required great operator precision. To address these issues, a placement guide system was specifically developed (described in Methods). The system has a standardized setup ensuring a consistent distance between electrodes. The database therefore contains recordings made in a very similar way to the preliminary study, with the same electrode configuration, the same electrodes and same recording device but with a slightly smaller inter-electrode distance (17.5 versus 21 mm). The system has a shorter electrode attachment time than for the one by one electrode attachment method from the preliminary study and the placement of the electrodes into the silicone backing can be done before a recording, shortening the setup time for an actual recording to around five minutes. This enables recordings to be performed when there is little time and reduces any inconveniences for participants and health professionals. The data from the preliminary study is not included in the database presented here.

All recordings were performed by the same researcher (A.A.) using the same protocol. The researcher (A.A.) stayed with the participants during recordings, recorded events first hand and monitored the equipment and electrode readouts continually. The tocodynamometer was recalibrated to 20 if readouts were zero. In the Embla A10 machine, an electrode that is unconnected or floating will give a signal which very quickly goes to saturation and is therefore easily recognized. If during a recording, an electrode gave a trace that was visually very unlike the traces of other electrodes or displayed values notably different from other values, it was pressed more firmly onto the abdomen to ensure connection to the skin. If all electrode traces seemed suboptimal (e.g., very noisy visually), the patient ground and reference electrode connections were inspected and the electrodes pressed more firmly onto the skin. In a few cases when suboptimal traces did not improve, the electrodes were reconnected and the recording then resumed. We therefore assume that although there may be parts of the data where the contact is faulty between the skin and the electrode, they are few and moreover they are easily recognizable.

Parts of the data have already been used for developing and analyzing various signal processing methods and have led to several publications^8,10–23^. The technical quality of the data has been thoroughly checked throughout this work and has never been found to be lacking.

## Usage Notes

Although EHG has become, in general, a well-proven technique, the EHG signals are known to be problematic in the sense that they are very low frequency and very low amplitude.

The individual monopolar signals contain the measurement of the electric potential at each site in the matrix as referenced to the patient ground, with the reference electrode as a reference for the patient ground circuit. The patient ground and reference electrodes were always more than 20 cm away from each electrode in the matrix (varying between participants and gestational ages) and positioned over the iliac crest where little electrical activity is suspected. No use was made of the other electrodes as reference, average or otherwise. The raw signals therefore contain everything that creates a potential difference between the monopolar electrode and the patient ground. This includes the maternal and fetal ECG and some EMG from striated muscles. The signal also contains artifacts related to movements of the participant, fetal movement, and even fetal respiration has been observed. Fetal hiccups can give periodic spikes that are clearly visible. Our system proved very robust to 50 Hz line noise and this was never observed during measurements. Neither active shielding nor active grounding was used in the recordings.

The setup resulted in a high level of electrode offset varying very slowly over time. There were considerable differences between electrodes during the same measurement sessions, with a typical value of the offset being several millivolts while the EHG signal is at the level of several tens of microvolts. Any processing of the signals could include high pass filtering with a very low cut-off frequency and/or a creation of a new reference using adjacent electrodes (i.e., bipolar or Laplacian).

During pregnancy recordings, the fetal or maternal heart rates were not recorded. During labor recordings, fetal heart rate was usually monitored by the clinical team, but was not collected as a part of the study.

The annotations and the tocograph complement each other. Some contractions that are present in the annotations are not obvious on the tocograph and some obvious contractions on the tocograph are not in the annotations. This explains in part why some recordings are without annotations. Noted fetal movement could last for differing amounts of time and participants did not always notify if they felt fetal movement. Sometimes fetal movement can be seen on the tocograph.

The first sample of a signal file is indexed with 1 in the .mat files but with 0 in the .dat files and so there is an index number discrepancy of one between the two file formats (.mat and .dat).

The MATLAB files contain the absolute raw units. Division by 131.068 gives the physical units in mV.

Even though some pregnancies ended in cesarean, the participant was on occasion already in spontaneous labor. These incidences are explained in the comments sections of the header files.

If a recording was close to birth, then the timing of the recording with regard to the birth is in the comments of the header file.

Our aim is to publish this database as is, without giving the user any detailed directions and thus encouraging open-minded exploitation of the data. Making raw data available is the best way to do this. There are however some pointers about how to make sense of the data which we would like to communicate to the users.

1. The data is sampled at 200 Hz. The EHG signals are generally assumed to be of very low frequency, from almost dc up to 3 Hz maximum. Decimating the raw signal (after low pass filtering) is advised, before or after creating a bipolar/multipolar signal or other de-noising. This will create more manageable files and it is often better to work with signals when the frequency of the signal to be observed is not as far away from the sampling frequency as in this case. This will also cut down calculation time in any complex analysis. Results have shown that decimation of this signal has little or even a positive effect on the performance of analysis methods^20^.
2. Please be aware that there are inherent imprecisions in the synchronization between the EHG recordings and the annotations and tocographs. There can be differences in when participants start to feel a contraction or fetal movements and so differences in when participants notify about events. Events were therefore occasionally approximated to the nearest whole minute. There are also delays internal to the tocodynamometers. Due to factors such as these, the inserted recording times on the tocographs and the annotation times may be up to±30 s from the actual recording or event times.
3. A user doing intensive work using these signals will have to develop an effective methodology to keep track of the signals, the way they have been pre-treated and the associated clinical parameters. Inspiration on how to organize such work can be sought in the structure of the SQL database framework which we developed^31^.

Users can view the data and annotations through two web interfaces: LightWAVE (a JavaScript viewer—www.physionet.org/lightwave) and ATM (Automated Teller Machine—www.physionet.org/cgi-bin/atm/ATM). ATM´s toolbox includes software that can convert WFDB signal files to text, CSV, EDF or .mat files and can show samples and annotations as text.

WAVE can also be used for viewing and analyzing the signals (www.physionet.org/physiotools/wfdb.shtml#WAVE). Annotations can be opened by using the ‘–a’ option with the wave WFDB application (www.physionet.org/physiotools/wag/wave-1.htm).

The WFDB Software Package can be used to work with the recordings (www.physionet.org/physiotools/wfdb.shtml). For example, the command ‘rdann -r *record name* -a atr -f 0’ can be used to read the annotation files (www.physionet.org/physiotools/wag/rdann-1.htm). The WFDB Toolbox for MATLAB and Octave can also be used to work with the recordings (www.physionet.org/physiotools/matlab/wfdb-app-matlab).

## Additional information

Table 1 is only available in the online version of this paper.

**How to cite this article:** Alexandersson, A. *et al.* The Icelandic 16-electrode electrohysterogram database. *Sci. Data* 2:150017 doi: 10.1038/sdata.2015.17 (2015).

## Supplementary Material



## Figures and Tables

**Table 1:** The clinical information from each of the 122 recordings in the Icelandic 16-electrode electrohysterogram database along with recording duration and whether or not the recording has a corresponding annotation file

**Filename**	**ID**	**Record type**	**Record number**	**Age (years)**	**BMI before pregnancy**	**BMI at recording**	**Gravidity**	**Parity**	**Previous caesarean**	**Placental position**	**Gestational age at recording (w/d)**	**Gestational age at delivery (w/d)**	**Mode of delivery**	**Synthetic oxytocin use in labour**	**Epidural during labour**	**Duration of recording (hh:mm:ss)**	**Annotation file**
ice001_l_1of1	ice001	labour	1	31	23.3	27.6	3	2	No	Fundus	39/3	39/3	Vaginal	No	No	0:08:20	No
ice002_p_1of3	ice002	pregnancy	1	38	20.7	25.9	4	1	No	Posterior	38/1	40/4	Vaginal	No	No	1:08:05	Yes
ice002_p_2of3	ice002	pregnancy	2	38	20.7	25.9	4	1	No	Posterior	39/1	40/4	Vaginal	No	No	1:02:10	Yes
ice002_p_3of3	ice002	pregnancy	3	38	20.7	26.4	4	1	No	Posterior	40/1	40/4	Vaginal	No	No	1:00:35	Yes
ice003_p_1of2	ice003	pregnancy	1	23	28.4	34.1	1	0	No	Anterior	37/6	40/0	Vaginal	No	No	1:01:10	Yes
ice003_p_2of2	ice003	pregnancy	2	23	28.4	34.6	1	0	No	Anterior	38/6	40/0	Vaginal	No	No	1:00:45	Yes
ice004_p_1of1	ice004	pregnancy	1	30	31.0	35.8	4	2	No	Posterior	36/5	41/3	Vaginal/Induction	No	No	1:06:10	Yes
ice005_p_1of3	ice005	pregnancy	1	28	29.0	31.2	2	0	No	Posterior	32/4	40/3	Vaginal	Yes	No	1:01:55	Yes
ice005_p_2of3	ice005	pregnancy	2	28	29.0	31.2	2	0	No	Posterior	34/6	40/3	Vaginal	Yes	No	0:59:20	Yes
ice005_p_3of3	ice005	pregnancy	3	28	29.0	31.6	2	0	No	Posterior	36/6	40/3	Vaginal	Yes	No	1:05:30	Yes
ice006_p_1of2	ice006	pregnancy	1	30	31.6	37.4	2	1	No	Posterior	36/1	37/6	Vaginal/Induction	Yes	No	1:02:10	Yes
ice006_p_2of2	ice006	pregnancy	2	30	31.6	37.6	2	1	No	Posterior	37/4	37/6	Vaginal/Induction	Yes	No	1:02:45	Yes
ice007_p_1of3	ice007	pregnancy	1	19	23.2	27.8	1	0	No	Posterior	32/5	41/2	Vaginal	No	No	1:06:40	Yes
ice007_p_2of3	ice007	pregnancy	2	19	23.2	28.1	1	0	No	Posterior	35/5	41/2	Vaginal	No	No	1:13:55	Yes
ice007_p_3of3	ice007	pregnancy	3	19	23.2	29.4	1	0	No	Posterior	37/5	41/2	Vaginal	No	No	1:01:50	Yes
ice008_p_1of4	ice008	pregnancy	1	26	20.9	25.3	5	2	No	Anterior	35/5	40/2	Vaginal	No	No	1:02:00	Yes
ice008_p_2of4	ice008	pregnancy	2	26	20.9	25.3	5	2	No	Anterior	37/6	40/2	Vaginal	No	No	1:00:55	Yes
ice008_p_3of4	ice008	pregnancy	3	26	20.9	25.0	5	2	No	Anterior	39/2	40/2	Vaginal	No	No	1:06:30	Yes
ice008_p_4of4	ice008	pregnancy	4	26	20.9	25.3	5	2	No	Anterior	40/1	40/2	Vaginal	No	No	1:05:30	Yes
ice009_p_1of2	ice009	pregnancy	1	24	22.8	25.3	2	1	No	Posterior	33/1	39/0	Vaginal	No	No	1:25:30	Yes
ice009_p_2of2	ice009	pregnancy	2	24	22.8	26.1	2	1	No	Posterior	38/1	39/0	Vaginal	No	No	0:49:35	Yes
ice010_p_1of3	ice010	pregnancy	1	22	19.1	23.0	1	0	No	Posterior	29/5	41/4	Vaginal	Yes	Yes	1:07:40	Yes
ice010_p_2of3	ice010	pregnancy	2	22	19.1	23.0	1	0	No	Posterior	31/5	41/4	Vaginal	Yes	Yes	1:02:00	Yes
ice010_p_3of3	ice010	pregnancy	3	22	19.1	23.8	1	0	No	Posterior	34/5	41/4	Vaginal	Yes	Yes	1:04:15	Yes
ice011_p_1of3	ice011	pregnancy	1	37	25.4	30.9	4	3	No	Anterior	36/0	40/0	Vaginal	Yes	No	1:06:10	Yes
ice011_p_2of3	ice011	pregnancy	2	37	25.4	31.1	4	3	No	Anterior	38/0	40/0	Vaginal	Yes	No	1:00:50	Yes
ice011_l_3of3	ice011	labour	3	37	25.4	31.8	4	3	No	Anterior	40/0	40/0	Vaginal	Yes	No	0:25:10	No
ice012_p_1of4	ice012	pregnancy	1	19	23.4	26.8	2	0	No	Posterior	33/0	40/6	Vaginal	No	Yes	1:01:05	Yes
ice012_p_2of4	ice012	pregnancy	2	19	23.4	26.8	2	0	No	Posterior	35/0	40/6	Vaginal	No	Yes	1:00:50	Yes
ice012_p_3of4	ice012	pregnancy	3	19	23.4	27.4	2	0	No	Posterior	37/0	40/6	Vaginal	No	Yes	1:00:55	Yes
ice012_p_4of4	ice012	pregnancy	4	19	23.4	27.8	2	0	No	Posterior	38/0	40/6	Vaginal	No	Yes	0:59:10	Yes
ice013_p_1of3	ice013	pregnancy	1	28	30.5	35.4	2	1	No	Posterior	34/4	40/4	Vaginal	No	No	1:02:20	Yes
ice013_p_2of3	ice013	pregnancy	2	28	30.5	36.0	2	1	No	Posterior	38/0	40/4	Vaginal	No	No	0:52:15	Yes
ice013_p_3of3	ice013	pregnancy	3	28	30.5	35.9	2	1	No	Posterior	39/0	40/4	Vaginal	No	No	1:03:40	Yes
ice014_p_1of3	ice014	pregnancy	1	33	28.1	32.2	3	2	No	Posterior	36/2	40/1	Vaginal	No	No	1:00:45	Yes
ice014_p_2of3	ice014	pregnancy	2	33	28.1	32.7	3	2	No	Posterior	38/2	40/1	Vaginal	No	No	1:02:35	Yes
ice014_p_3of3	ice014	pregnancy	3	33	28.1	32.8	3	2	No	Posterior	39/2	40/1	Vaginal	No	No	0:51:10	Yes
ice015_p_1of1	ice015	pregnancy	1	23	25.1	30.7	1	0	No	Anterior	36/4	38/3	Emergency caesarean due to other than slow progress	No	No	1:14:55	Yes
ice016_l_1of1	ice016	labour	1	20	23.9	30.5	3	0	No	Anterior	39/3	39/3	Vaginal	Yes	Yes	0:50:55	Yes
ice017_p_1of3	ice017	pregnancy	1	31	19.5	24.4	2	1	No	Anterior	36/5	42/1	Vaginal/Induction	Yes	No	0:46:50	Yes
ice017_p_2of3	ice017	pregnancy	2	31	19.5	25.2	2	1	No	Anterior	39/3	42/1	Vaginal/Induction	Yes	No	1:00:40	Yes
ice017_p_3of3	ice017	pregnancy	3	31	19.5	25.3	2	1	No	Anterior	40/6	42/1	Vaginal/Induction	Yes	No	0:19:10	Yes
ice018_p_1of1	ice018	pregnancy	1	37	24.1	28.9	4	2	Yes	Posterior	38/1	38/4	Elective caesarean	No	No	1:01:10	Yes
ice019_p_1of1	ice019	pregnancy	1	29	21.1	25.2	4	2	No	Fundus	40/2	41/1	Vaginal	No	No	0:55:15	No
ice020_l_1of1	ice020	labour	1	24	20.9	27.2	1	0	No	Posterior/Lateral left	41/5	41/5	Vaginal	Yes	Yes	1:04:10	No
ice021_p_1of3	ice021	pregnancy	1	27	25.4	30.4	1	0	No	Anterior	35/3	40/2	Vaginal	Yes	Yes	1:03:40	Yes
ice021_p_2of3	ice021	pregnancy	2	27	25.4	30.4	1	0	No	Anterior	36/6	40/2	Vaginal	Yes	Yes	1:03:00	Yes
ice021_p_3of3	ice021	pregnancy	3	27	25.4	30.4	1	0	No	Anterior	37/6	40/2	Vaginal	Yes	Yes	1:02:35	Yes
ice022_p_1of1	ice022	pregnancy	1	21	19.6	24.4	1	0	No	Anterior	34/2	38/0	Vaginal	No	No	1:08:10	Yes
ice023_p_1of1	ice023	pregnancy	1	29	31.6	33.5	2	1	No	Posterior	32/4	40/5	Vaginal	Yes	Yes	1:04:50	Yes
ice024_l_1of1	ice024	labour	1	35	38.6	42.2	2	1	No	Posterior	39/6	39/6	Vaginal	No	No	0:25:20	Yes
ice025_l_1of1	ice025	labour	1	27	27.3	30.8	2	0	No	Anterior	41/2	41/2	Vaginal	Yes	Yes	0:51:40	Yes
ice026_p_1of4	ice026	pregnancy	1	25	26.2	28.5	1	0	No	Anterior	35/0	39/5	Vaginal	Yes	No	0:52:45	Yes
ice026_p_2of4	ice026	pregnancy	2	25	26.2	28.6	1	0	No	Anterior	37/0	39/5	Vaginal	Yes	No	1:00:55	Yes
ice026_p_3of4	ice026	pregnancy	3	25	26.2	28.9	1	0	No	Anterior	38/0	39/5	Vaginal	Yes	No	1:00:50	Yes
ice026_p_4of4	ice026	pregnancy	4	25	26.2	29.0	1	0	No	Anterior	39/0	39/5	Vaginal	Yes	No	1:05:15	Yes
ice027_p_1of7	ice027	pregnancy	1	31	31.1	34.5	7	2	No	Anterior	29/5	38/1	Vaginal	Yes	No	0:56:45	No
ice027_p_2of7	ice027	pregnancy	2	31	31.1	35.0	7	2	No	Anterior	33/3	38/1	Vaginal	Yes	No	1:01:15	Yes
ice027_p_3of7	ice027	pregnancy	3	31	31.1	35.2	7	2	No	Anterior	34/5	38/1	Vaginal	Yes	No	1:00:25	No
ice027_p_4of7	ice027	pregnancy	4	31	31.1	35.5	7	2	No	Anterior	35/5	38/1	Vaginal	Yes	No	1:00:10	Yes
ice027_p_5of7	ice027	pregnancy	5	31	31.1	35.2	7	2	No	Anterior	36/5	38/1	Vaginal	Yes	No	1:00:25	Yes
ice027_p_6of7	ice027	pregnancy	6	31	31.1	35.5	7	2	No	Anterior	37/5	38/1	Vaginal	Yes	No	1:01:40	Yes
ice027_l_7of7	ice027	labour	7	31	31.1	35.5	7	2	No	Anterior	38/1	38/1	Vaginal	Yes	No	0:32:00	No
ice028_p_1of3	ice028	pregnancy	1	31	21.6	24.9	4	1	No	Posterior	34/1	38/0	Vaginal	No	No	1:03:30	Yes
ice028_p_2of3	ice028	pregnancy	2	31	21.6	25.3	4	1	No	Posterior	36/1	38/0	Vaginal	No	No	1:00:30	Yes
ice028_p_3of3	ice028	pregnancy	3	31	21.6	25.7	4	1	No	Posterior	37/1	38/0	Vaginal	No	No	1:06:20	Yes
ice029_p_1of2	ice029	pregnancy	1	25	24.5	28.3	3	2	No	Anterior/Superior	32/5	35/3	Vaginal/Induction	Yes	Yes	1:00:20	Yes
ice029_p_2of2	ice029	pregnancy	2	25	24.5	28.8	3	2	No	Anterior/Superior	34/5	35/3	Vaginal/Induction	Yes	Yes	1:00:40	Yes
ice030_p_1of4	ice030	pregnancy	1	33	20.1	25.6	2	1	Yes	Posterior/Superior	33/6	37/0	Vaginal	Yes	Yes	1:01:25	No
ice030_p_2of4	ice030	pregnancy	2	33	20.1	26.0	2	1	Yes	Posterior/Superior	35/6	37/0	Vaginal	Yes	Yes	1:04:25	Yes
ice030_p_3of4	ice030	pregnancy	3	33	20.1	26.4	2	1	Yes	Posterior/Superior	36/6	37/0	Vaginal	Yes	Yes	1:01:00	Yes
ice030_l_4of4	ice030	labour	4	33	20.1	26.4	2	1	Yes	Posterior/Superior	37/0	37/0	Vaginal	Yes	Yes	0:31:30	No
ice031_p_1of3	ice031	pregnancy	1	38	35.0	35.9	6	3	Yes	Anterior	36/1	39/0	Vaginal	Yes	No	1:00:15	Yes
ice031_p_2of3	ice031	pregnancy	2	38	35.0	35.6	6	3	Yes	Anterior	38/3	39/0	Vaginal	Yes	No	1:00:35	Yes
ice031_l_3of3	ice031	labour	3	38	35.0	35.8	6	3	Yes	Anterior	39/0	39/0	Vaginal	Yes	No	0:37:55	Yes
ice032_p_1of5	ice032	pregnancy	1	31	26.0	28.7	1	0	No	Posterior	31/6	40/1	Vaginal	No	No	0:34:10	Yes
ice032_p_2of5	ice032	pregnancy	2	31	26.0	28.7	1	0	No	Posterior	34/0	40/1	Vaginal	No	No	1:01:15	Yes
ice032_p_3of5	ice032	pregnancy	3	31	26.0	29.4	1	0	No	Posterior	36/0	40/1	Vaginal	No	No	1:07:00	Yes
ice032_p_4of5	ice032	pregnancy	4	31	26.0	29.8	1	0	No	Posterior	37/2	40/1	Vaginal	No	No	1:08:25	Yes
ice032_p_5of5	ice032	pregnancy	5	31	26.0	30.1	1	0	No	Posterior	40/0	40/1	Vaginal	No	No	1:00:35	Yes
ice033_p_1of1	ice033	pregnancy	1	20	20.4	24.1	3	0	No	Anterior	33/2	39/1	Vaginal	Yes	Yes	1:00:45	Yes
ice034_p_1of5	ice034	pregnancy	1	36	22.1	24.9	3	2	No	Anterior/High	30/3	38/5	Vaginal/Induction	Yes	Yes	1:01:45	Yes
ice034_p_2of5	ice034	pregnancy	2	36	22.1	25.2	3	2	No	Anterior/High	32/3	38/5	Vaginal/Induction	Yes	Yes	1:04:35	Yes
ice034_p_3of5	ice034	pregnancy	3	36	22.1	25.7	3	2	No	Anterior/High	35/3	38/5	Vaginal/Induction	Yes	Yes	1:03:20	Yes
ice034_p_4of5	ice034	pregnancy	4	36	22.1	25.8	3	2	No	Anterior/High	37/5	38/5	Vaginal/Induction	Yes	Yes	0:50:00	Yes
ice034_l_5of5	ice034	labour	5	36	22.1	25.8	3	2	No	Anterior/High	38/5	38/5	Vaginal/Induction	Yes	Yes	0:36:00	No
ice035_p_1of3	ice035	pregnancy	1	34	34.3	36.3	3	2	Yes	Anterior/High	32/0	39/0	Elective caesarean	No	No	1:00:25	Yes
ice035_p_2of3	ice035	pregnancy	2	34	34.3	36.5	3	2	Yes	Anterior/High	34/0	39/0	Elective caesarean	No	No	1:00:25	Yes
ice035_p_3of3	ice035	pregnancy	3	34	34.3	37.0	3	2	Yes	Anterior/High	36/0	39/0	Elective caesarean	No	No	1:00:15	Yes
ice036_p_1of6	ice036	pregnancy	1	39	31.0	35.5	5	2	No	Anterior/High	31/6	41/1	Emergency caesarean due to other than slow progress	No	No	1:00:30	Yes
ice036_p_2of6	ice036	pregnancy	2	39	31.0	36.2	5	2	No	Anterior/High	34/2	41/1	Emergency caesarean due to other than slow progress	No	No	0:58:40	Yes
ice036_p_3of6	ice036	pregnancy	3	39	31.0	35.5	5	2	No	Anterior/High	37/3	41/1	Emergency caesarean due to other than slow progress	No	No	1:04:55	Yes
ice036_p_4of6	ice036	pregnancy	4	39	31.0	35.8	5	2	No	Anterior/High	38/3	41/1	Emergency caesarean due to other than slow progress	No	No	1:03:15	Yes
ice036_p_5of6	ice036	pregnancy	5	39	31.0	35.8	5	2	No	Anterior/High	39/3	41/1	Emergency caesarean due to other than slow progress	No	No	0:43:50	Yes
ice036_p_6of6	ice036	pregnancy	6	39	31.0	35.8	5	2	No	Anterior/High	40/2	41/1	Emergency caesarean due to other than slow progress	No	No	1:02:10	Yes
ice037_p_1of2	ice037	pregnancy	1	33	26.6	31.9	2	1	Yes	Posterior/High	33/5	38/5	Emergency caesarean due to other than slow progress	No	No	1:03:35	Yes
ice037_p_2of2	ice037	pregnancy	2	33	26.6	32.6	2	1	Yes	Posterior/High	36/5	38/5	Emergency caesarean due to other than slow progress	No	No	0:59:20	Yes
ice038_p_1of1	ice038	pregnancy	1	29	28.4	32.2	4	1	Yes	Anterior/High	33/3	37/2	Elective caesarean	No	No	1:00:40	Yes
ice039_p_1of6	ice039	pregnancy	1	34	29.8	29.9	1	0	No	Anterior/High	31/4	40/0	Vaginal	No	No	1:02:20	Yes
ice039_p_2of6	ice039	pregnancy	2	34	29.8	29.6	1	0	No	Anterior/High	33/4	40/0	Vaginal	No	No	0:58:45	Yes
ice039_p_3of6	ice039	pregnancy	3	34	29.8	30.0	1	0	No	Anterior/High	35/5	40/0	Vaginal	No	No	1:01:50	Yes
ice039_p_4of6	ice039	pregnancy	4	34	29.8	30.1	1	0	No	Anterior/High	37/2	40/0	Vaginal	No	No	1:02:10	Yes
ice039_p_5of6	ice039	pregnancy	5	34	29.8	30.5	1	0	No	Anterior/High	38/2	40/0	Vaginal	No	No	1:02:30	Yes
ice039_p_6of6	ice039	pregnancy	6	34	29.8	30.3	1	0	No	Anterior/High	39/2	40/0	Vaginal	No	No	0:56:20	Yes
ice040_p_1of5	ice040	pregnancy	1	39	20.7	28.2	2	0	No	Posterior/High	36/5	41/6	Vaginal/Induction	Yes	No	1:03:00	Yes
ice040_p_2of5	ice040	pregnancy	2	39	20.7	28.9	2	0	No	Posterior/High	37/5	41/6	Vaginal/Induction	Yes	No	0:52:20	Yes
ice040_p_3of5	ice040	pregnancy	3	39	20.7	29.4	2	0	No	Posterior/High	38/5	41/6	Vaginal/Induction	Yes	No	1:00:35	Yes
ice040_p_4of5	ice040	pregnancy	4	39	20.7	29.7	2	0	No	Posterior/High	39/5	41/6	Vaginal/Induction	Yes	No	1:01:55	Yes
ice040_p_5of5	ice040	pregnancy	5	39	20.7	29.7	2	0	No	Posterior/High	40/6	41/6	Vaginal/Induction	Yes	No	1:01:20	Yes
ice041_p_1of2	ice041	pregnancy	1	26	33.9	37.6	3	1	No	Anterior/Right	36/0	38/5	Vaginal/Induction	No	No	1:02:30	Yes
ice041_p_2of2	ice041	pregnancy	2	26	33.9	37.9	3	1	No	Anterior/Right	36/5	38/5	Vaginal/Induction	No	No	1:03:05	Yes
ice042_p_1of2	ice042	pregnancy	1	25	20.3	22.6	2	0	No	Anterior/High	34/4	40/3	Vaginal	Yes	Yes	1:00:40	Yes
ice042_p_2of2	ice042	pregnancy	2	25	20.3	22.9	2	0	No	Anterior/High	38/2	40/3	Vaginal	Yes	Yes	0:46:55	Yes
ice043_p_1of2	ice043	pregnancy	1	25	24.1	26.2	2	0	No	Anterior	33/2	40/5	Emergency caesarean due to other than slow progress	No	No	1:06:15	No
ice043_p_2of2	ice043	pregnancy	2	25	24.1	26.9	2	0	No	Anterior	36/4	40/5	Emergency caesarean due to other than slow progress	No	No	1:01:15	Yes
ice044_p_1of3	ice044	pregnancy	1	28	23.2	28.4	1	0	No	Anterior/High	36/3	40/6	Vaginal	Yes	No	1:01:55	Yes
ice044_p_2of3	ice044	pregnancy	2	28	23.2	28.7	1	0	No	Anterior/High	38/5	40/6	Vaginal	Yes	No	1:04:10	Yes
ice044_p_3of3	ice044	pregnancy	3	28	23.2	29.1	1	0	No	Anterior/High	40/5	40/6	Vaginal	Yes	No	0:58:30	Yes
ice045_p_1of4	ice045	pregnancy	1	26	24.7	29.4	4	1	No	Anterior/High	33/2	40/0	Vaginal	No	No	1:06:45	Yes
ice045_p_2of4	ice045	pregnancy	2	26	24.7	29.8	4	1	No	Anterior/High	35/6	40/0	Vaginal	No	No	1:05:55	Yes
ice045_p_3of4	ice045	pregnancy	3	26	24.7	30.1	4	1	No	Anterior/High	37/3	40/0	Vaginal	No	No	1:07:15	Yes
ice045_p_4of4	ice045	pregnancy	4	26	24.7	30.5	4	1	No	Anterior/High	39/4	40/0	Vaginal	No	No	1:11:00	Yes
